# EHMTI-0062. Expression of fractalkine (CX3CL1) and fractalkine receptor (CX3CR1) in the trigeminal ganglia: implications for craniofacial nociception

**DOI:** 10.1186/1129-2377-15-S1-F7

**Published:** 2014-09-18

**Authors:** P Gazerani, BE Cairns, XD Dong

**Affiliations:** 1 Department of Health Science and Technology Center for Sensory-Motor Interaction, Aalborg University, Aalborg, Denmark; 2 Faculty of Pharmaceutical Sciences, the University of British Columbia, Vancouver, Canada

## Introduction

Evidence indicates that fractalkine (CX3CL1) and its receptor CX3CR1 are involved in the pathogenesis of pain. Within the central nervous system, CX3CL1 is expressed by neurons and CX3CR1 by microglia. This unique pattern of expression has supported the idea of neuron to glia communication, which is thought to be disregulated under pathological pain conditions. The presence of CX3CL1 and CX3CR1 in trigeminal ganglion (TG) neurons and satellite glial cells (SGCs) and their contribution to nociceptive signal transmission have not been well documented.

## Aims

To investigate the expression of CX3CL1 and CX3CR1 in naive rat trigeminal ganglia.

## Methods

Trigeminal ganglia were isolated from one adult male and one adult female Sprague-Dawley rat. Immunohistochemistry was performed on 5 ganglia sections per rat to determine the expression of CX3CL1 and CX3CR1 on trigeminal neurons and SGCs. ImageJ was used for data analysis.

## Results

CX3CL expression was observed in only 3% of TG neurons (diameter: 9-46 μm) and was rarely observed in SGCs. In contrast, 39% of TG neurons (diameter: 5-58 μm) expressed CX3CR1. CX3CR1 was also expressed in trigeminal SGCs, many of which surrounded CX3CR1-positive TG neurons.

## Conclusions

CX3CR1 is expressed by TG neurons of widely varying sizes and by many SGCs. CX3CL1 immunoreactivity was only rarely identified in TG neurons or SGCs. Cross talk between neurons and SGCs through fractalkine signaling does not appear likely under normal conditions. Increased expression of CX3CL1 in the TG may occur following craniofacial inflammation or injury, which is currently under investigation in our laboratory.

No conflict of interest.

